# Social Determinants of Health and Diabetes-Related Distress in Patients With Insulin-Dependent Type 2 Diabetes: Cross-sectional, Mixed Methods Approach

**DOI:** 10.2196/40164

**Published:** 2022-10-12

**Authors:** Natalie K Levy, Agnes Park, Daniela Solis, Lu Hu, Aisha T Langford, Binhuan Wang, Erin S Rogers

**Affiliations:** 1 Department of Medicine New York University Grossman School of Medicine New York, NY United States; 2 Department of Population Health New York University Grossman School of Medicine New York, NY United States

**Keywords:** social determinants of health, income, socioeconomic, cross sectional, insulin, diabetic, HbA_1c_, barrier, diabetes-related distress, type 2 diabetes, ambulatory care, healthcare, health care, distress, epidemiology, T2DM, diabetes

## Abstract

**Background:**

Social determinants of health (SDOH) refer to the social, economic, and psychosocial conditions that influence health. Lower levels of SDOH factors including income, education, and employment are associated with a higher prevalence of diabetes, poorer glycemic control, and increased diabetes-related mortality. Few studies have conducted a comprehensive evaluation of multiple SDOH factors in a population with type 2 diabetes mellitus (T2DM).

**Objective:**

This study aimed to identify the range of SDOH challenges—including diabetes-related distress—that impact patients with insulin-dependent diabetes at an urban safety-net clinic using the 5-domain SDOH framework developed by the Healthy People 2020 initiative.

**Methods:**

The pilot study used a cross-sectional, mixed methods approach. Participants were recruited from 3 programs within a general internal medicine clinic that provides ambulatory care for patients with uncontrolled T2DM. We administered an investigator-developed SDOH survey based on the Healthy People 2020 framework and the validated Diabetes Distress Scale (DDS), which assesses 4 domains of diabetes-related distress. One-on-one interviews were conducted to gain in-depth information about challenges.

**Results:**

In total, 57 participants had an average hemoglobin A_1c_ level of 11.0% (SD 2.6%). Overall, 92% (52/57) of participants had a barrier in at least one SDOH domain. SDOH challenges were most commonly reported in the domain of Health and Health Care (84%, 48/57), followed by Economic Stability (54%, n=31), Neighborhood and Built Environment (53%, n=30), Education and Health Literacy (47%, n=27), and Social and Community context (37%, n=21). The mean overall DDS score was 2.09 (SD 0.84), where scores of ≥2 indicate distress. Further, 79% (45/57) of participants had at least moderate diabetes-related distress in one of the 4 DDS domains. General themes that emerged from participant interviews included job interference with healthy behaviors, concerns about burdening others, challenges communicating with providers, and difficulty getting appointments in a timely manner.

**Conclusions:**

We found high levels of SDOH barriers across all 5 domains of the Center for Disease Control and Prevention’s Healthy People 2020 framework, including significant levels of diabetes-related distress. Future programs to address SDOH barriers in patients with uncontrolled insulin-dependent diabetes should consider screening for and focusing on a wide range of challenges.

## Introduction

Social determinants of health (SDOH) are broadly defined as the circumstances in which people are born, live, and work [1,2]; they include the social, economic, and psychosocial conditions that influence health [1-4]. In 2010, the US Department of Health and Human Services published the goals for its Healthy People 2020 (HP2020) initiative, which included a new section on SDOH; this framework organizes key issues into one of 5 domains: Health and Health Care, Economic Stability, Neighborhood and Built Environment, Education, and Social and Community Context [1].

Over 30 million Americans are estimated to have diabetes, and another 84 million are estimated to have prediabetes [5,6]. Diabetes increases the risk of heart disease, stroke, kidney failure, blindness, and lower limb amputation [5,6]. In 2017, the estimated cost of diagnosed diabetes in the United States was US $327 billion [5,6]. Studies examining the connections between SDOH and diabetes have shown that lower levels of SDOH factors including income, education, and employment are associated with a higher prevalence of diabetes [7], increased diabetes-related mortality [8], and poorer glycemic control [9-11].

To date, most diabetes-related SDOH literature has generally assessed a narrow subset of SDOH challenges at one time [12-15]. A 2014 systematic review evaluating the impact of SDOH on outcomes for type 2 diabetes mellitus (T2DM) included articles whose focus was mostly clustered around a single SDOH domain: 28 focused on Health and Health Care, 17 on Social and Community Context, 11 on Economic Stability, 3 on Neighborhood and Built Environment, and 1 on Education [12]. Similarly, studies included in a 2021 review by the American Diabetes Association mainly assessed single SDOH domains [15]. It is less often that a single study evaluates multiple SDOH factors in the same population [9,14].

While not an individual category in the HP2020 SDOH framework, psychosocial distress is also considered an SDOH factor [2,3,16]. Diabetes-related psychosocial distress (hereinafter referred to as “diabetes-related distress” [DRD]) is associated with poor glycemic control and self-care [2,17-19]. Research estimates the prevalence of DRD in the United States to be 18%-48% [17,19-22]. However, the average hemoglobin A_1c_ (HbA_1c_) levels in the populations studied ranged from 6.7% to 9.9% [17,19-22], where a level of <8% is generally considered to indicate good control and that of ≥11% is considered poor for individuals with diabetes. The prevalence of DRD in a population of patients with T2DM with poorer glycemic control is less studied.

To fill SDOH-related knowledge gaps for patient populations with T2DM, this pilot study sought to distinguish itself in several ways. First, this study evaluated SDOH barriers across a broad range of SDOH domains. Second, instead of looking at larger upstream SDOH factors (eg, income, education, and employment), this study sought to evaluate how SDOH challenges affect patients and their diabetes management on a day-to-day basis. Finally, we evaluated SDOH barriers and DRD in a population likely to have poorer glycemic control than previously reported.

## Methods

### Ethical Considerations

Study approval was granted by the institutional review board of the New York University Grossman School of Medicine (NYUGSoM; s17-01553). All participants provided written informed consent in English or Spanish in the presence of a bilingual study team member.

### Study Setting

This study was conducted at Bellevue Hospital, New York City, New York, which is affiliated with NYUGSoM [23]. Bellevue is the oldest public hospital in the United States and is part of NYC Health + Hospitals, the largest municipal health care system in the United States. Bellevue provides safety-net care to a diverse population of over 30,000 patients—approximately one-third of whom are uninsured—through its Adult Primary Care Center.

### Participants and Recruitment

From January 2018 to November 2018, patients with insulin-dependent T2DM and an HbA_1c_ level of ≥7.0% and who speak English or Spanish were recruited from 3 settings within Bellevue Hospital’s Adult Primary Care Center: (1) the High A1C Clinic, a referral program imbedded within primary care that focuses on the management of patients with poorly controlled diabetes; (2) the Diabetes Group Medical Visit, a 4-week, multidisciplinary program that provides comprehensive self-management education; and (3) the Mobile Insulin Titration Intervention (MITI) Program, a telehealth service that uses basic SMS text messages and phone calls to adjust basal insulin remotely [24-26]. To address selection bias, all physicians of the High A1C Clinic and Diabetes Group Medical Visit as well as the MITI enrollment coordinator were informed about the study and eligibility criteria (ie, insulin-dependent T2DM, an HbA_1c_ level of ≥7.0%, and speaking English or Spanish). Physicians and the MITI enrollment coordinator were asked to provide information about the study to every patient who met the eligibility criteria. Frequent reminders were provided each week during physician huddles to encourage recruitment efforts.

Upon receiving informed consent, bilingual research assistants administered the study questionnaires and conducted the study interview. All study procedures were completed in one sitting, and participants were provided US $30 in cash upon completion of study activities.

### Study Instruments

#### The SDOH Questionnaire

##### Overview

We developed a 15-item questionnaire that assessed for SDOH factors across the 5 HP2020 SDOH domains that could affect the health and well-being of patients with T2DM. The majority of questions were adapted from the Protocol for Responding to and Assessing Patients’ Assets, Risks, and Experiences (PRAPARE) survey, a standardized tool to screen for SDOH [27]. The SDOH questionnaire also examined participants’ levels of physical activity. Questions were categorized into 6 sections.

##### Economic Stability

One question was adapted from PRAPARE to assess if participants had been unable to pay for an essential item when they really needed it in the past year. In total, 12 items were listed in the choices, including food, medication, and medical visits.

##### Education and Health Literacy

The following questions were adapted from a health literacy scale developed at the University of California, Los Angeles [28]: “How often do you have trouble: 1) explaining health concerns to a doctor or nurse; 2) understanding what a doctor or nurse says; 3) understanding written instructions on medication labels; 4) completing medical forms?” Participants had the option to respond with “often,” “sometimes,” and “never.”

##### Social and Community Context

Two questions were adapted from the PRAPARE survey: “How often do you see or talk to people that you care about and feel close to?” and “Who are the people you speak to when you are feeling stressed?”

##### Health and Health Care

One question was adapted from the PRAPARE survey to assess whether lack of transportation prevented the patient from consulting a doctor within the past year. We assessed for factors beyond the PRAPARE survey, such as “lack of insurance,” “can’t get an appointment,” and “hard to miss work.”

##### Neighborhood and Built Environment

Two questions were adapted from the PRAPARE survey to assess for housing situation and housing security. Additional questions were added by the research team to assess for the availability and quality of fruits and vegetables in the neighborhood and the availability of safe places to exercise.

##### Physical Activity

We adapted questions from the US National Health Interview Survey (NHIS) [29] to ask if participants engaged in physical activity outside of any paid job and the frequency, duration, and vigorousness of the physical activity.

#### The DRD Scale

DRD was measured using the validated 17-question Diabetes Distress Scale (DDS) [30,31]. The DDS measures distress in 4 areas: Emotional Burden, Regimen Distress, Interpersonal Distress, and Physician Distress. Responses are scored on a Likert scale. Total scores of 2-2.9 indicate moderate distress and those of ≥3 indicate severe distress. For this study, a total score of ≥2 was considered the threshold for describing patients as having distress, as advised by the DDS and prior studies [18,21,32,33].

#### Semistructured Interview Guide

The first 31 participants who completed the 2 study questionnaires were also asked to complete a semistructured interview. This sample size was determined by the number of participants who were interviewed until thematic saturation was reached. The research team developed an interview guide to gather in-depth information about the SDOH challenges patients reported on the SDOH questionnaire and DDS. The questions were written to allow participants to expand upon the SDOH challenges they identified and how those challenges impacted their diabetes.

### Electronic Medical Record Data Abstraction

Sociodemographic data and HbA_1c_ values were abstracted from electronic medical records. HbA_1c_ values were the most recent values taken within 3 months from the date of enrollment.

### Statistical Analyses

The SDOH quantitative data are reported as the number and percentage of participants who had challenges within each HP2020 domain, its subcategories, and the number and percentage of participants who had at least one barrier across a certain number of HP2020 domains. DDS data are reported such that for each individual DDS domain, the average score as well as the number and percentage of patients having scores in at least the moderate (≥2) and the severe (≥3) range are reported. These data are also reported by age, language, and gender. The number and percentage of patients who have at least moderate distress across a certain number of domains is also reported. Spearman and Pearson correlations were used to measure the level of association between HbA_1c_ values and SDOH data (number of challenge domains, presence or lack of challenges in individual domains), HbA_1c_ and DDS data (overall and domain DDS scores, number of domains with distress, and number of domains with severe distress), and overall DDS scores and number of SDOH challenge domains.

For quantitative data, descriptive statistics were used to summarize demographic characteristics and other factors. Continuous variables (eg, age and HbA_1c_) are described as means and SDs, and categorical variables (such as gender) are summarized as frequencies and percentages. For qualitative data, analyses were conducted on Atlas.ti (version 8.1; ATLAS.ti Scientific Software Development GmbH). Interviews were transcribed verbatim, and Spanish-language transcripts were translated into English. In total, 3 investigators used both deductive and inductive (grounded theory) approaches to code the interviews. They created an initial codebook that included 5 main SDOH domains from the interview guide, domain and code definitions, and inclusion and exclusion criteria. They then independently coded 4 transcripts, followed by discussions of coding agreement and disagreement and an updating of the codebook to include open coding of SDOH subcodes. When the codebook was complete, they independently coded the remaining transcripts, with co-coding of every fifth transcript to ensure intercoder reliability. Once coding was complete, they met to identify themes.

## Results

### Participant Characteristics

In total, 61 patients were screened for eligibility. Of those, 60 were eligible to participate. In total, 3 declined enrollment owing to scheduling difficulties. In total, 57 patients participated in the study. The mean age of participants was 54.8 (SD 12.1) years, 56% (n=32) were Hispanic, 28% (n=16) were uninsured, and the average HbA_1c_ level was 11.0% (SD 2.6%). Additional characteristics are provided in Table 1.

**Table 1:** Participant demographics (N=57).

Demographics		Values
Age (years), mean (SD)		54.8 (12.1)
**Gender, n (%)**		
	Male	35 (61)
	Female	22 (39)
**Language**		
	English	33 (58)
	Spanish	24 (42)
**Race and ethnicity, n (%)**		
	White	6 (11)
	Black	14 (25)
	Hispanic	32 (56)
	Asian	5 (9)
**Insurance status, n (%)**		
	Medicaid	26 (46)
	Medicare	6 (11)
	Medicaid + Medicare	9 (16)
	Uninsured	16 (28)
Hemoglobin A_1c_ levels (%), mean (SD)		11.0 (2.6)

### SDOH Questionnaire Data

Data were obtained on the percentage of patients who had challenges within each of the 5 major HP2020 domains in the past year. All participants provided responses to every question on the SDOH questionnaire. Overall, 84% (48/57) of participants had a barrier in Health and Health Care in the past year, including 49% (28/57) who were unable to get an appointment. In total, 56% (32/57) of participants had a barrier in Economic Stability and reported ≥1 instance when they were unable to pay for an essential item when it was truly needed. This included 23% (13/57) of participants who were unable to pay rent and 23% (13/57) who were unable to pay for healthy food. Overall, 53% (30/57) of participants had a barrier in their Neighborhood and Built Environment including 32% (18/57) of participants with no or only a very small number of stores that sell produce in their neighborhood and 16% (9/57) having no safe place to exercise. Overall, 47% (27/57) of participants had an Education and Health Literacy challenge, including 42% (24/57) of those who had difficulty explaining their health concerns to a doctor and 18% (10/57) of those who had trouble reading a medication label. Approximately one-third of participants had challenges in the domain of Social and Community Context, including 37% (21/57) of participants who shared that they speak to or see someone who they care about ≥2 times per week, and 30% (17/57) of them speak to no one when stressed. The presence or lack of challenges in the domain of Health and Health Care was significantly correlated with HbA_1c_ values (ρ=0.32, *P*=.02). Correlation coefficients for all other domains were <0.5 and nonsignificant (*P*>.05).

Across the 5 HP2020 domains, 92% (52/57) of patients had a barrier in at least one domain, 73% (42/57) in at least 2 domains, 62% (35/57) in at least 3 domains, and 44% (25/57) in at least 4 domains. A further breakdown of challenges within each main domain are provided in Table 2. There was no significant correlation between the number of SDOH challenge domains and HbA_1c_ values (ρ=0.23, *P*=.09).

**Table 2:** Frequency of social determinants of health barriers reported by the study participants (N=57).

Domain				Participants, n (%)
**Health and Health Care**				
	**Had ≥1 barrier to health care access in the past year**		84 (48)	
		Could not get appointment	49 (28)	
		Forgot the appointment	40 (23)	
		Difficult to miss work	21 (12)	
		Cost or lack of insurance	18 (10)	
		Transportation difficulties	14 (8)	
		Childcare difficulties	7 (4)	
		Other	12 (7)	
**Economic Stability**				
	**Unable to pay for ≥1 essential items in the past year**		56 (32)	
		Rent	23 (13)	
		Healthy food	23 (13)	
		Any food	18 (10)	
		Medical visit	16 (9)	
		Medical supplies	16 (9)	
		Utilities	16 (9)	
		Phone	14 (8)	
		Medication	14 (8)	
		Clothing	12 (7)	
		Mortgage	4 (2)	
		Childcare	0 (0)	
**Neighborhood and Built Environment**				
	**Has ≥1 issue with the built environment**		53 (30)	
		Worried about losing housing	19 (11)	
		No stores or only a small number of stores that sell produce in the neighborhood	30 (17)	
		Unsatisfied with the quality of produce available in the neighborhood	9 (5)	
		Do not have safe places to exercise in the neighborhood	16 (9)	
**Education and Health Literacy**				
	Has ≥1 issue with health literacy		47 (27)	
	Has difficulty explaining health concerns to a doctor or nurse		42 (24)	
	Has difficulty understanding what a doctor or nurse is saying		19 (11)	
	Has difficulty filling out medical forms		19 (11)	
	Has difficulty understanding medication labels		18 (10)	
**Social and Community Context**				
	Receives social support ≤2 times a week		37 (21)	
	Has nobody to speak to when stressed		30 (17)	

### DDS Data

DDS scores were calculated for 56 participants and are listed in Table 3. One participant did not complete the DDS owing to challenges with understanding the questions. The largest individual domain of DRD on the DDS was Emotional Burden (mean 2.62, SD 1.37), with 64% of participants having at least moderate distress (ie, score≥2) and 34% having severe distress (ie, score≥3). The second most common distress domain was Regimen Distress (mean 2.41, SD 1.13), with 57% (32/57) of participants having at least moderate distress and 30% having severe distress. The areas of Interpersonal Distress and Physician Distress were present but were generally less of a challenge for participants, with means of 1.68 (SD 0.91) and 1.33 (SD 0.55), respectively. Emotional Burden and Regimen Distress remained the largest domains of DRD after analysis by age, language, and gender. As with SDOH, participants had DRD across multiple domains: 79% (45/57) of participants had at least moderate distress in ≥1 domain, 52% (30/57) in ≥2 domains, and 36% (21/57) in ≥3 domains. There were no significant correlations between HbA_1c_ value and overall score (*r*=–0.006, *P=*.96) and individual domain scores.

**Table 3:** Mean participant Diabetes Distress Scale scores^a^ and categorization by age, language, and gender (N=56^b^.).

Variables	Emotional Burden	Physician Distress	Regimen Distress	Interpersonal Distress	Overall Score
Score, mean (SD)	2.62 (1.37)	1.33 (0.55)	2.41 (1.13)	1.68 (0.91)	2.09 (0.84)
Ages 29-40 years (n=8), mean	3.10	1.38	2.34	2.50	2.39
Ages 41-59 years (n=27), mean	2.71	1.50	2.49	1.52	2.15
Age ≥60 years (n=21), mean	2.34	1.18	2.24	1.57	1.89
English speakers (n=32), mean	2.51	1.27	2.41	1.74	1.98
Spanish speakers (n=24), mean	2.62	1.42	2.42	1.60	2.04
Male (n=35), mean	2.47	1.29	2.13	1.69	1.95
Female (n=21), mean	2.87	1.40	2.85	1.64	2.32
Participants with at least moderate distress, n (%)	36 (64)	8 (14)	32 (57)	21 (38)	26 (46)

^a^2.0-2.9: moderate distress; ≥3: high distress

^b^One participant was unable to complete the Diabetes Distress Scale.

### Participant Interview Data

Themes identified from participant interviews were grouped by SDOH domain and are presented alongside representative quotes in Table 4.

Within the domain of Economic Stability, participants regularly experienced difficulty affording items that were necessary to manage their diabetes and care for their health. Consequences included delayed treatment and the foregoing of certain items, despite participants recognizing the impact these would have on their health. Participants also reported challenges stemming from their jobs. Many worked long hours (eg, late nights, 7 days a week) and had unpredictable schedules. As a result, they had little time or energy to cook and exercise. In addition, some participants reported difficulty taking time off from work, which impacted their ability to attend doctor’s visits.

Within the Social and Community Context domain, more than half of participants reported feeling like they did not have a support system for their diabetes. In particular, these individuals felt that they lacked people in their personal lives who understood what diabetes was or who they could talk to about their experience living with diabetes. Some participants noted that their family or friends impeded healthy eating efforts by offering them unhealthy foods. Participants who had friends and family members with diabetes shared that they provided a source of knowledge and support for coping, regularly checked in with the participant, and helped participants with their diabetes-related care. Many participants described experiencing a personal emotional toll from their diabetes. Finally, participants did not want to burden their loves ones with their diabetes and instead wanted to try to deal with their health on their own.

Within the domain of Neighborhood and Built Environment*,* several participants reported having limited access to healthy foods in their neighborhood, particularly fresh vegetables. Participants were frequently exposed to advertisements for fast foods, which made it difficult to resist unhealthy eating behaviors.

Within the Education and Health Literacy domain, some participants reported difficulty communicating with their providers. This included the following experiences with providers: the providers spoke too quickly, spoke too coldly, used medical terminology that the participants did not understand, or did not fully explain the participants’ condition and how to take care of it.

Lastly, within the Health and Health Care domain, participants reported difficulty getting health care appointments. This impacted their diabetes because their physicians wanted them to return for follow-up visits every 2-3 months but it often took much longer to get an appointment. Participants also experienced significant delays and frustrations owing to perceived disorganization and a lack of communication among different departments within the health care system. 

**Table 4:** Social determinants of health and themes in the Diabetes Distress Scale identified during semistructured interviews.

Social determinants of health domain and themes			Example quotes
**Economic Instability**			
	Difficulty affording health-related needs	“Sometimes the way you eat is based on survival because it’s like $10 for a salad right? But it’s like $5 for a steak sandwich. So it’s like when you making financial choices and you trying to eat based on your budget. It conflicts because you know that you can’t have a Philly cheesesteak because of your diabetes but then again, you gotta eat something…So it’s about survival then.” (M117) “My income is not enough. I have rent, food, everything, so I just cannot pay for the medical visit.” (M109)	
	Job interferes with healthy behavior	“I just don’t have the time [to exercise]. I work 11-12 hours daily, every day. I come home tired, I take a shower and go to sleep.” (M123) Interviewer: What are some challenges you have in being able to see your doctor for your diabetes? “Financially, permission from work, sometimes I don’t have money. It’s all together.” (M114)	
**Social and Community Context**			
	Limited or no support for diabetes-related health	“I had some family staying with me and because the type of food [they were cooking], I think it contributed to [my blood sugar getting out of control].” (M109) “Sometimes, people [don’t] understand diabetic people. Sometimes, I’m visit my friend, she’s having a birthday and gives me cake. Sometimes [she says], ‘You eat, you eat.’…Sometimes, people [don’t] understand.” (M105)	
	Loved ones with diabetes are supportive of diabetes-related health	“If I feel a concern, I would just always talk to my father, because he’s been with diabetes and he’ll give me an example [of what to do]… ‘cause he’s been through the same thing.” (M106) “I get a call from my cousin every day, so sometimes she’ll ask me if I did check my sugar.” (M107)	
	Participant feels an emotional toll associated with having diabetes	“You know you’re supposed to be doing better but it’s like, you know, how can you? It does put you in a—um, a clouded mental space. […] Like ,you know you be beat. You don’t wanna beat yourself up but it’s like, how do you make better choices?” Interviewer: What is the emotion you feel the most often when you struggle with your diabetes? “Frustration and sadness.” (M104)	
	Participant does not want to burden others	“I have got plenty of [family], but, you know, sometimes everybody has their own thing to do. You don’t want to burden nobody with your things so try to get your thing over by yourself.” (M118)	
**Neighborhood and Built Environment**			
	Environmental exposure to unhealthy food	“[It’s harder] when watching TV and sometimes when commercials come on with food…psychologically it makes me hungry and I start eating the wrong things when I see commercials.” (M119)	
	Limited access to healthy food in the neighborhood	Interviewer: What are some reasons that you find eating healthy challenging? “It’s just finding a place to buy the vegetables.” (M108)	
**Education and Health Literacy**			
	Challenges communicating with providers	“There are some words I still do not know. It might [help if my doctor would] explain things more slowly or that is when he speak sometimes talking is very fast and very cold, indifferent.” (M116)	
	Language discordance	“The diabetes doctor for example speaks not an ounce of Spanish. But she also doesn’t use the translator phone.” (M135)	
**Health and Health Care**			
	Cannot get appointments in a timely manner	“I haven’t seen my primary in five months, so that’s half a year gone by there. You call and there’s no appointments available.” (M130)	
	Disorganization of the health care system	“They shouldn’t be giving me appointments that are too close together, because then I might miss one or literally run from one appointment to the next and get late. 15 minutes late, they don’t want to see you. I’ve seen people get frustrated…extremely to the point where they’re yelling and screaming.” (M130)	

## Discussion

### Principal Findings

This study used a mixed methods approach to identify challenges across the 5 domains of the Healthy People 2020 SDOH framework and the presence of DRD, measured by the validated DDS, in patients with insulin-dependent T2DM seeking care at a safety-net hospital. Participants had substantial SDOH barriers, which created regular and significant challenges for participants wanting to better manage their diabetes. Data demonstrated that the challenges were extensive, impacting nearly every aspect of participants’ day-to-day lives—from difficulties affording health-related needs to encountering limited understanding of diabetes among loved ones—as well as their interactions with the health care system and health care teams. Given the association between glycemic control and DRD, it is noteworthy, but not surprising, that 46% of participants also had DRD. To our knowledge, our participant sample has the highest average HbA_1c_ levels (mean 11.0%, SD 2.6%) out of any cohort for which DRD prevalence has been reported [17-22,32-37].

The lack of significant correlations between HbA_1c_ levels and SDOH data (with the exception of the Health and Health Care SDOH domain) and DDS data may, in part, be due to the fact that all participants in our study were insulin-dependent. Generally, patients with T2DM are started on insulin when hyperglycemia is severe or when other therapies have not been successful at lowering blood sugar levels to a well-controlled range [38]. It is possible that correlations are more significant up until the point when diabetes is severe enough to warrant insulin (ie, insulin may subsequently lower blood sugar to more optimal levels but the conditions that contributed to needing insulin may still persist).

Prior studies have often focused on big-picture “upstream” SDOH factors (eg, education, poverty, and employment). For example, data from the 1990-2000 US NHIS combined with that from the NHIS Linked Mortality Files through 2002 showed that having less than a high school education and having a family income below the poverty line were each associated with a 2-fold higher mortality from diabetes, compared to adults with a college degree or higher or with those with the highest family income, respectively [8]. Data from the 2015 NHIS found that having less than a high school education, having a family income of less than US $35,000 a year, and being “not employed but having worked previously” were each associated with about twice the risk of having diabetes compared to those who had graduated college, had a family income of US $100,000 or greater, or who were employed full time [7]. Building upon these data, our study looked within the broad categories and drilled down to focus on the day-to-day effects of these SDOH challenges that are not typically reported. Our findings shed light on the potential mechanisms by which the broad SDOH factors studied previously may impact one’s ability to manage their diabetes. For example, poverty can reduce one’s ability to afford healthy food and live in safe neighborhoods with access to fresh produce, while increasing one’s reliance on busy safety-net health systems with limited appointment availability.

By comprehensively assessing for SDOH challenges, we found that patients had barriers across several domains simultaneously: 73% (42/57) of patients having barriers across ≥2 domains, 62% (35/57) having barriers across ≥3 domains, and 44% (25/57) having barriers across ≥4 domains. These findings suggest that prior studies that assessed a single or small number of SDOH domains likely underestimated the prevalence of SDOH challenges in their participant samples. Clinical settings that rely on single-domain SDOH screeners (eg, food insecurity and risk for homelessness) also risk underestimating the social needs felt by a significant portion of their patients. Therefore, future SDOH research and SDOH-driven clinical program development should use comprehensive screening tools, such as the PRAPARE tool [27], the Accountable Health Communities Health-Related Social Needs Screening Tool [39], or the Health Leads Social Needs Screening Tool [40].

This work has clinical significance. As the movement to recognize the importance of SDOH grows stronger, not only will policy makers need to understand and focus on the big-picture SDOH (eg, poverty and education level) but local health systems gearing up to help patients with T2DM overcome such barriers need to know how these upstream SDOH challenges effects patients’ daily lives. This work details such “day to day” challenges. In addition, the evaluation of barriers in multiple domains reinforces that patients with T2DM can have a wide range of concomitant challenges. Diabetes team members charged with helping at-risk patients will need to be prepared to address challenges that span across multiple SDOH domains. Future research is needed to help health systems identify best practices in addressing SDOH challenges identified in our study, which are not typically within the scope of health care, such as helping patients overcome work-related barriers to healthy eating or exercising (eg, working long hours), obtaining healthy food when living in neighborhoods with no fresh produce, or engaging loved ones in discussions about the need for support. In addition, this work can serve as an example to the entire diabetes team, that high levels of DRD may accompany such patients. Given that both SDOH and DRD are associated with glycemic control, both need to be recognized and addressed as part of care for the whole patient.

### Limitations

There are several limitations to this study. First, it was conducted at a single site—an ambulatory clinic at a safety-net hospital in New York City—and thus may not be generalizable to other settings. Second, in an effort to target a population with a high likelihood of SDOH challenges and DRD, the study sample was limited to patients with insulin-dependent T2DM. Our findings may not generalize to other patient groups, and we are not able to determine whether SDOH barriers are more severe in our study sample than in patients with T2DM who are not insulin-dependent. Third, owing to the study’s cross-sectional design, we are unable to assess changes in participants’ SDOH profiles over a period of time or establish relationships between SDOH and diabetes control. Lastly, while efforts were made to reduce bias in our recruitment methods and all participants responded to all questionnaire items, except for one who did not complete the DDS, our participants who opted to be part of our study may be different from those who did not participate.

### Conclusions

This pilot study found high levels of SDOH barriers across all 5 domains of the Center for Disease Control and Prevention’s Health People 2020 SDOH framework, including significant levels of DRD. Future programs to address SDOH barriers in patients with uncontrolled insulin-dependent diabetes in safety-net programs are needed and should be designed to screen for and address a wide range of challenges.

## Abbreviations

DDS: Diabetes Distress Scale
DRD: diabetes-related distress
HbA_1c_: hemoglobin A_1c_
HP2020: Healthy People 2020
MITI: Mobile Insulin Titration Intervention
NHIS: National Health Interview Survey
NYUGSoM: New York University Grossman School of Medicine
PRAPARE: Protocol for Responding to and Assessing Patients’ Assets, Risks, and Experiences
SDOH: social determinants of health
T2DM: type 2 diabetes mellitus
